# Health Benefits of Epigallocatechin Gallate and Forskolin with a Special Emphasis on Glaucoma and Other Retinal Diseases

**DOI:** 10.3390/medicina60121957

**Published:** 2024-11-27

**Authors:** Dario Rusciano

**Affiliations:** Fidia Ophthalmics, 95124 Catania, Italy; drusciano55@gmail.com

**Keywords:** epigallocatechin gallate, forskolin, eye, retinal disease, glaucoma, macular degeneration, diabetic retinopathy

## Abstract

This review highlights the therapeutic potential of epigallocatechin gallate (EGCG) and forskolin in managing retinal diseases, with a focus on glaucoma, age-related macular degeneration (AMD), and diabetic retinopathy. EGCG, a potent polyphenol from green tea, exhibits significant antioxidant, anti-inflammatory, and neuroprotective effects, making it a promising candidate for reducing oxidative stress and inflammation in ocular tissues. Forskolin, a diterpene from Coleus forskohlii, increases cyclic AMP (cAMP) levels, which helps lower intraocular pressure (IOP) and provides neuroprotection. Both compounds target critical pathways involved in retinal disease progression, including oxidative stress, mitochondrial dysfunction, and inflammation, offering complementary therapeutic benefits. This review consolidates preclinical and clinical studies, highlighting the potential of EGCG and forskolin as adjunctive or alternative treatments for retinal diseases. Future research should explore the synergistic effects of these compounds, particularly in combination therapies aimed at addressing multiple pathogenic mechanisms in retinal health.

## 1. Introduction

**Figure 1:**
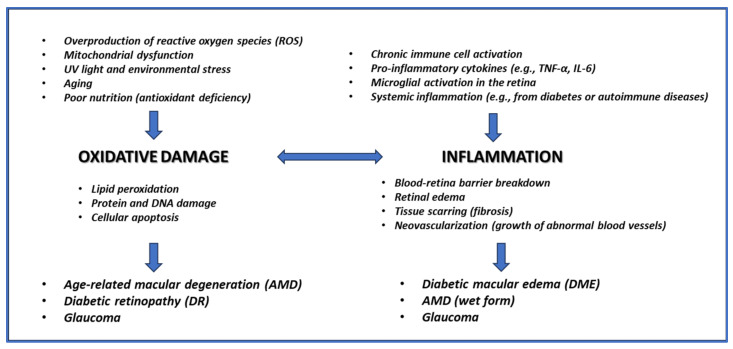
Main causes and effects of oxidative damage and inflammation leading to the development of the main retinal diseases.

Oxidative stress and inflammation are central to the development and progression of glaucoma and other eye diseases [1,2]. In glaucoma—a slowly progressing neurodegenerative disease affecting the optic nerve [3]—the buildup of reactive oxygen species (ROS) damages ocular tissues, particularly the trabecular meshwork and retinal ganglion cells (RGCs), disrupting cellular homeostasis and contributing to increased intraocular pressure (IOP) [4]. This leads to optic nerve degeneration and vision loss. Chronic inflammation further aggravates this damage by activating pathways that degrade ocular tissue integrity [4]. In conditions like age-related macular degeneration (AMD) and diabetic retinopathy, oxidative stress also drives disease progression by damaging retinal cells and promoting harmful deposits (e.g., drusen in AMD) or impairing the blood–retinal barrier in diabetic retinopathy [5]. Inflammation accelerates cellular degeneration and contributes to retinal and optic nerve damage in these conditions [5]. Together, oxidative stress and inflammation create a cycle of tissue damage, emphasizing the need for therapeutic strategies targeting these processes to slow or prevent disease progression [4] (Figure 1).

**Figure 2:**
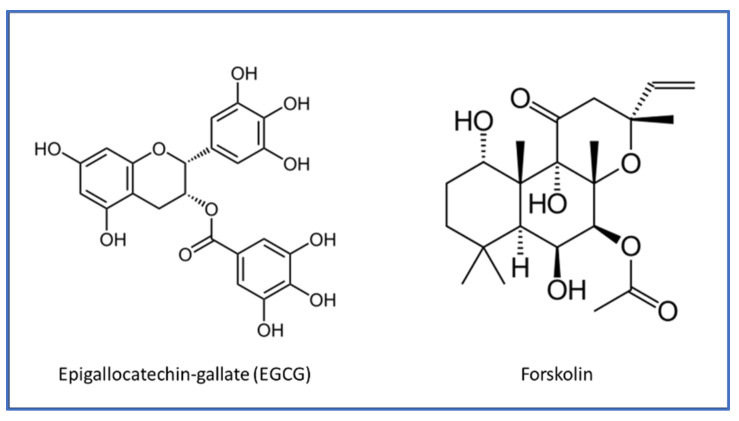
Chemical structure of EGCG and forskolin.

The use of antioxidants and anti-inflammatory treatments, particularly through dietary supplements, has gained attention in managing glaucoma and other ophthalmic diseases. Antioxidants such as vitamins C and E, omega-3 fatty acids, and plant-derived compounds like flavonoids and carotenoids help neutralize ROS and reduce oxidative stress, a key factor in these diseases [6]. In glaucoma, antioxidant supplementation can protect the optic nerve and RGCs, potentially slowing vision loss [7]. Similarly, in AMD [8] and diabetic retinopathy [9,10], antioxidants have been shown to protect retinal cells from oxidative injury, reducing disease progression. Anti-inflammatory supplements, such as omega-3 fatty acids and curcumin, modulate inflammatory pathways that contribute to ocular damage. These supplements can reduce chronic inflammation, protecting the trabecular meshwork in glaucoma [11,12] and preventing retinal inflammation in AMD and diabetic retinopathy [13,14,15]. Although more research is needed to standardize treatment protocols, antioxidants and anti-inflammatory supplements are promising adjuncts to traditional medical and surgical treatments for glaucoma and other eye diseases. Combining epigallocatechin gallate (EGCG) and forskolin in a single supplement might hold significant promise for preventing and treating various eye diseases. EGCG, a powerful antioxidant and anti-inflammatory compound found abundantly in green tea, has strong neuroprotective effects, particularly in addressing oxidative stress and inflammation in ocular pathologies [16]. Forskolin, a natural extract from Coleus forskohlii, lowers IOP by increasing cAMP levels in ocular tissues, which is critical in managing glaucoma [17]. The synergistic effects of these compounds offer a comprehensive approach to eye health, targeting oxidative stress, inflammation, and IOP reduction (Figure 2). Their combination could enhance current treatments, providing a multifaceted defense against progressive eye diseases, making it a compelling candidate for further research and development in ocular health supplements.

This review explores the scientific foundation for the synergy between EGCG and forskolin and assesses the benefits and safety of incorporating them into vision and eye health supplements. 

## 2. EGCG Biochemical Properties and Health Effects

### 2.1. Antioxidant

EGCG is well known for its strong antioxidant effects. By neutralizing ROS, EGCG helps mitigate oxidative stress, a key factor in the development of various organ pathologies, including ocular diseases like glaucoma, age-related macular degeneration (AMD), and diabetic retinopathy [18]. In addition to its antioxidative properties, EGCG has significant anti-inflammatory effects by inhibiting key mediators such as NF-κB and COX-2, reducing chronic inflammation in eye diseases [19].

### 2.2. Neuroprotection

EGCG also offers remarkable neuroprotective properties. It protects mitochondria from dysfunction [20], a crucial factor in glaucoma where mitochondrial impairment plays a pivotal role [21]. EGCG reduces neuroinflammation, enhancing communication between the nervous and immune systems, thus preserving neurological functions [22]. It also inhibits the aggregation of neurotoxic proteins like amyloid-beta and tau, known to contribute to conditions such as Alzheimer’s disease, thus protecting against neural degeneration [23].

### 2.3. Antitumoral

The therapeutic potential of EGCG was first identified in cancer research, where it demonstrated significant anti-cancer properties through multiple mechanisms. It inhibits cancer cell growth by inducing programmed cell death (apoptosis) and halting cell proliferation. EGCG suppresses angiogenesis, essential for tumor growth, by downregulating vascular endothelial growth factor (VEGF) and angiogenic factors [24,25]. Additionally, EGCG modulates matrix metalloproteinases (MMPs), enzymes responsible for degrading the extracellular matrix, thereby preventing cancer cell invasion and metastasis [26]. EGCG also enhances the efficacy of cancer therapies by overcoming drug resistance, making it a potent candidate for combination treatments [27].

EGCG affects several cellular signaling pathways, such as PI3K/Akt, MAPK, and NF-κB, which are critical for cancer cell survival and proliferation [28,29]. Furthermore, it exerts epigenetic modifications, including DNA methylation and histone modification, to activate tumor suppressor genes and inhibit oncogenes [30]. In estrogen-dependent cancers like breast cancer, EGCG modulates estrogen receptor signaling [31]. It has also been shown to inhibit androgen receptors in prostate cancer, thus reducing cancer cell proliferation and promoting apoptosis [32]. Additionally, EGCG targets lung cancer by inhibiting the cell cycle and metastasis [33], and it exhibits similar antiproliferative effects in colorectal cancer [34] and leukemia [35,36]. Overall, EGCG’s multi-targeted approach, influencing both signaling pathways and gene expression, highlights its broad therapeutic potential in cancer management.

### 2.4. Metabolism and Weight Management

EGCG shares with forskolin an interesting role in metabolism and weight management, which may also benefit ocular health by reducing the risk of related eye conditions. EGCG enhances fat oxidation, converting stored fat into energy. Studies show that consuming green tea extract, rich in EGCG, can increase fat oxidation during exercise, leading to weight loss over time [37,38]. It boosts metabolism by increasing thermogenesis, a process that burns more calories even at rest, especially when combined with caffeine, also present in green tea [39,40]. Several studies suggest EGCG contributes to modest weight loss, particularly in combination with green tea extract, by increasing fat oxidation and metabolic rate [41,42]. EGCG specifically targets visceral fat, a harmful fat linked to metabolic diseases such as type 2 diabetes and cardiovascular disease [38,43].

EGCG may also reduce appetite by influencing gut hormones associated with food intake, supporting weight loss efforts [44,45]. There is evidence that it increases feelings of fullness, leading to reduced calorie intake [46]. EGCG improves insulin sensitivity, helping regulate blood sugar levels and reducing the risk of metabolic syndrome, a condition characterized by high blood pressure, high blood sugar, excess body fat, and abnormal cholesterol levels [47,48,49]. EGCG may influence the expression of genes related to obesity [50]. Accordingly, EGCG may inhibit the formation of new fat cells (adipogenesis) while promoting the breakdown of existing fat cells (lipolysis) [51]. Emerging evidence suggests that EGCG combined with caffeine may positively affect the gut microbiota, which plays a role in energy balance, fat storage, and overall metabolism [52]. A healthier gut microbiome is linked to a lower risk of obesity. The anti-inflammatory properties of EGCG may help reduce chronic inflammation associated with obesity and metabolic syndrome, improving metabolic health and reducing weight gain [53].

The role of metabolic health is increasingly recognized as a crucial factor in the onset and progression of retinal diseases, especially diabetic retinopathy and AMD. Dysregulated glucose metabolism and obesity-related oxidative stress are known contributors to retinal damage. EGCG’s ability to enhance fat oxidation and improve insulin sensitivity may therefore have significant implications for retinal health, as these metabolic improvements can reduce the overall inflammatory and oxidative burden on the retina.

In conclusion, EGCG’s diverse biochemical properties make it a compelling therapeutic agent across multiple diseases, including metabolic syndrome, neurodegenerative conditions, cancers, and—as we will see below—ocular disorders. Its potential to mitigate oxidative stress, inflammation, and cell death highlights its value as a candidate for future treatments targeting complex health challenges.

## 3. EGCG Efficacy on Retinal Diseases

As metabolic dysfunction plays a central role in several retinal diseases, particularly diabetic retinopathy, the systemic effects of EGCG on glucose regulation and fat metabolism can directly influence retinal health. By improving insulin sensitivity and reducing visceral fat, EGCG may mitigate the chronic inflammation and oxidative stress that contribute to retinal cell damage in these diseases.

In ocular health, EGCG shows continued promise. By protecting RGCs from apoptosis, it helps preserve vision in glaucoma. Its ability to modulate cellular signaling pathways supports cell survival and prevents apoptosis, while its antiangiogenic properties benefit conditions such as diabetic retinopathy and AMD. EGCG’s metal ion chelation abilities enhance its neuroprotective and antioxidant functions, protecting ocular tissues from oxidative damage caused by metals [54,55].

### 3.1. Mitochondrial Dysfunction

Pioneering preclinical studies have demonstrated the efficacy of EGCG (shown to be more potent than Trolox) in treating oxidative eye diseases, particularly glaucoma and AMD [56]. These conditions are driven by oxidative stress and mitochondrial dysfunction, which lead to the degeneration of retinal cells, including RGCs and photoreceptors [57].

Mitochondrial dysfunction is a key factor in many retinal diseases. Photobiomodulation (PBM) therapy, which uses non-invasive light therapy, has gained attention for its potential to alleviate this dysfunction. PBM enhances mitochondrial function, reduces oxidative stress, and promotes cell survival. For retinal diseases, low-level lasers or light-emitting diodes (LEDs) are typically directed toward the eye in the red or near-infrared spectrum (600 to 1000 nm) for 5 to 30 min. PBM sessions can be conducted daily or several times per week, preferably accompanied by protective eyewear [58,59]. Studies show that PBM promotes mitochondrial biogenesis by increasing the expression of key transcription factors like PGC-1α and NRF-1, enhancing mitochondrial respiration and improving adenosine triphosphate (ATP) production [60,61]. This effect is crucial in high-energy-demand tissues such as the retina and optic nerve. In conjunction with PBM, EGCG enhances mitochondrial biogenesis and respiratory efficiency [62,63], synergistically improving ATP production and reducing oxidative stress and ROS levels in ocular tissues, which amplifies the protective benefits against retinal cell apoptosis and neurodegeneration.

### 3.2. Oxidative Damage

In glaucoma, RGCs are particularly vulnerable to oxidative damage due to their energy demands and reliance on mitochondrial function. Consistently with its strong antioxidant power, EGCG also reduces ischemia-induced oxidative damage in hypertensive glaucoma models, preserving retinal cell function [64], even when given by the oral route [65]. In DR, oxidative stress is exacerbated by hyperglycemia, and EGCG helps neutralize ROS, protecting the retina from further damage [66], also through the activation of PKCα as a neuronal survival factor [67]. Additionally, EGCG inhibits COX-2, an enzyme that in AMD promotes inflammation and oxidative damage in retinal pigment epithelial (RPE) cells [68].

### 3.3. Glutamate Excitotoxicity

EGCG also plays a crucial role in preventing glutamate excitotoxicity in glaucoma by mitigating the harmful effects of excessive glutamate accumulation and the overactivation of NMDA receptors, which lead to calcium overload, oxidative stress, and mitochondrial dysfunction in RGCs. Glutamate excitotoxicity triggers a domino effect, where dying RGCs release more glutamate into the extracellular space, further overstimulating adjacent RGCs and exacerbating cell death through a bystander effect [69]. Additionally, EGCG helps regulate calcium homeostasis, thus preventing mitochondrial dysfunction, and enhances the clearance of excess glutamate by supporting glial cell function [70]. Through these mechanisms, EGCG interrupts the cycle of RGC degeneration, potentially halting the progression of glaucoma and protecting against vision loss [71]. Moreover, glutamate excitotoxicity is not limited to glaucoma. It plays a role in several neurodegenerative diseases, including multiple sclerosis (MS) [72], Alzheimer’s [73], Parkinson’s [74], and amyotrophic lateral sclerosis (ALS) [75]. Therefore, EGCG could potentially be beneficial also in the treatment of these other neurodegenerative diseases [76,77,78,79].

### 3.4. Inflammation

EGCG also exerts strong anti-inflammatory effects. In glaucoma, it suppresses inflammation by inhibiting key inflammatory pathways such as NF-κB and restoring Th1/Th2 cytokine balance, thus mitigating optic nerve damage [80]. In AMD, it reduces inflammation by downregulating pro-inflammatory cytokines like IL-6 and TNF-α, limiting the damage caused by choroidal neovascularization (CNV) [81]. EGCG’s ability to inhibit JNK and ERK pathways also reduces cell death in AMD [82]. In DR, EGCG inhibits the ROS/TXNIP/NLRP3 inflammasome pathway, preserving retinal cell function by reducing inflammation caused by high glucose levels [83]. This anti-inflammatory action helps to preserve retinal cell function and prevents further degeneration, especially in Müller cells, which play a critical role in maintaining retinal homeostasis.

### 3.5. Angiogenesis

EGCG’s antiangiogenic properties are crucial in managing conditions like AMD and DR, where abnormal blood vessel growth contributes to vision loss. Angiogenesis, driven by overexpression of VEGF, is a key factor in these diseases. EGCG inhibits VEGF production by modulating the PI3K/AKT and MAPK/ERK pathways, preventing abnormal blood vessel formation [84,85]. It also reduces MMP-9 activity, which breaks down the extracellular matrix, further inhibiting angiogenesis in DR [86]. In AMD, EGCG reduces VEGFA expression and downregulates the HIF-1α/VEGF/VEGFR2 pathway, preventing choroidal neovascularization and limiting vision loss [81]. On the other hand, EGCG also exerts neuroprotective effects by activating endothelial nitric oxide synthase (eNOS), enhancing nitric oxide (NO) production, and improving blood perfusion. Through the phosphorylation of pathways like PI3K/Akt, EGCG stimulates eNOS, increasing NO levels and promoting vasodilation, which improves vascular function and blood flow, particularly to neural tissues such as the retina [54]. This enhanced perfusion supports better oxygen and nutrient delivery, crucial in conditions like glaucoma where impaired circulation contributes to RGC degeneration. EGCG’s antioxidant properties reduce oxidative stress, protecting eNOS from uncoupling and preventing neuronal damage by scavenging reactive oxygen species (ROS) and maintaining mitochondrial health [87]. In animal models of diabetic retinopathy, EGCG has been shown to reduce blood–retinal barrier breakdown, improve retinal function, and restore the expression of tight junction proteins like claudin-1, which are critical for maintaining the integrity of the blood–retinal barrier [88]. These findings underscore the broad protective effects of EGCG in preventing both vascular and neurodegenerative complications in DR.

### 3.6. Fibrosis

EGCG’s antiproliferative effects are particularly useful in preventing scarring and fibrosis in conditions like glaucoma filtration surgery. EGCG inhibits myofibroblast transformation, which contributes to fibrosis, by blocking the TGF-β1/Smad signaling pathway [89]. It also prevents the migration and adhesion of RPE cells involved in the formation of epiretinal membranes in AMD, through inhibition of PDGF-beta receptor activity and downstream signaling pathways [90].

### 3.7. Neuroprotection

EGCG has neuroprotective properties, particularly in inhibiting apoptosis in RGCs and retinal pigment epithelial cells, which are crucial for treating glaucoma and AMD. EGCG protects RGCs from apoptosis caused by ischemia–reperfusion injury and elevated IOP by inhibiting caspase activation, a key apoptosis pathway [64,91]. EGCG also protects RGCs from damage after optic nerve axotomy by regulating apoptosis-related genes. Axotomy increases pro-apoptotic proteins nNOS and Bax, but EGCG treatment reduces their levels, preventing excessive cell death. EGCG also boosts anti-apoptotic signaling via enhanced activation of ERK 1/2 and Akt pathways. Blocking these pathways weakens EGCG’s protective effect, showing its role in promoting RGC survival by inhibiting pro-apoptotic signals and activating cell survival mechanisms [92]. Additionally, EGCG boosts NAD production by activating NMNAT2, an enzyme critical for neuron survival, which further protects RGCs from degeneration [93,94]. In AMD, EGCG reduces UVB-induced apoptosis in retinal pigment epithelial cells by modulating the JNK and ERK pathways [82]. ER stress contributes to AMD by triggering prolonged unfolded protein response (UPR) and apoptosis. EGCG protects mouse RPE cells by restoring calcium homeostasis, reducing ROS production, and inhibiting apoptosis-related markers like CHOP and cleaved caspases. It also enhances Akt and PTEN signaling to regulate UPR, suggesting its therapeutic potential in AMD by balancing ER stress and preventing cell death [95].

### 3.8. Autophagy

EGCG also restores autophagic activity, which is impaired in retinal diseases like AMD and glaucoma. Autophagy dysfunction in AMD leads to the accumulation of cellular debris in RPE cells, contributing to oxidative damage. EGCG restores autophagic flux by modulating the mTOR pathway, preventing apoptosis in RPE cells [96]. In glaucoma, EGCG enhances autophagic activity in Tenon’s fibroblasts, reducing fibrosis after glaucoma filtration surgery by promoting autophagosome formation and reducing p62 levels [97]. Autophagy is also disrupted in DR due to hyperglycemia. In diabetic conditions, autophagic dysfunction leads to the accumulation of cellular debris, promoting inflammation and cell death. EGCG restores autophagic activity by enhancing autophagosome formation and lysosomal function, protecting Müller cells and retinal pigment epithelial (RPE) cells from apoptosis. This mechanism is crucial in preventing retinal neurodegeneration, which is a key feature in the early stages of DR [98]. By reestablishing proper autophagy, EGCG prevents the buildup of toxic cellular components, thereby protecting retinal cells from damage.

### 3.9. AGEs

In addition to its antiangiogenic, antioxidant, and autophagy-regulating properties, EGCG inhibits the aldose reductase enzyme, a key player in the polyol pathway, which is activated under hyperglycemic conditions. This pathway contributes to the formation of advanced glycation end products (AGEs), which exacerbate oxidative stress and inflammation. EGCG’s inhibition of aldose reductase reduces the accumulation of AGEs and minimizes their damaging effects on retinal cells [99]. This is particularly important as aldose reductase activity is a major contributor to microvascular complications in DR, including retinal damage.

### 3.10. Trabecular Meshwork

Another mechanism explaining the role of EGCG in glaucoma is linked to its effects on trabecular meshwork (TM) cells. It is known that dysfunction of TM cells increases the resistance to AH outflow, leading to IOP elevation. This dysfunction is linked to endoplasmic reticulum (ER) stress in TM cells [100]. EGCG has been shown to reduce ER stress, also including human and porcine TM cells exposed in vitro to tunicamycin, a compound that induces ER stress [101]. The results of this study show that EGCG improves cell viability and significantly reduces the expression of key ER stress markers (ATF4, HSPA5, and DDIT3) in both human and porcine TM cells. These findings suggest that EGCG could protect TM cells from ER stress, offering a potential therapeutic approach to control IOP in glaucoma patients.

### 3.11. Clinical Evidence

Clinical studies support EGCG’s efficacy in improving retinal function in glaucoma patients. In a randomized, placebo-controlled trial, oral EGCG supplementation increased pattern-evoked electroretinogram (PERG) amplitudes in open-angle glaucoma patients, indicating improved retinal function [102]. Another study demonstrated that green tea extract and EGCG significantly reduced IOP in healthy volunteers [103], likely due to its relaxing effect on the trabecular meshwork [101].

**Figure 3:**
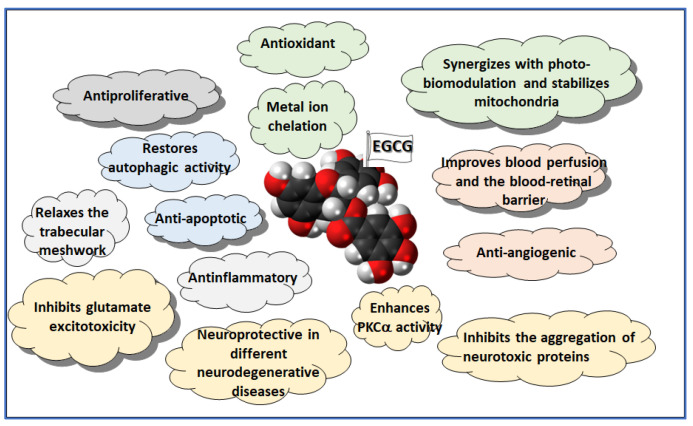
Pleiotropic effects of EGCG (the main catechin present in green tea) relevant to ophthalmic diseases. Related activities are highlighted with the same color: yellow for neuroprotection, pink for vasoactive effects, green for antioxidant activity, light blue for cell survival, light grey for IOP control, dark grey for antifibrotic effect.

Overall, EGCG’s antioxidant, anti-inflammatory, neuroprotective, and antiangiogenic properties make it a promising treatment for oxidative and inflammatory eye diseases like glaucoma, AMD, and DR (Figure 3). Its ability to be administered orally with minimal side effects, along with ongoing improvements in its delivery methods, underscores its potential as a safe and effective option for preserving vision and preventing disease progression.

## 4. Forskolin Biochemical Properties and Health Effects

Forskolin is a natural compound extracted from the roots of the Coleus forskohlii plant, a member of the mint family. Its primary mechanism of action involves activating adenylate cyclase, an enzyme that converts ATP to cyclic AMP (cAMP). Forskolin has been traditionally used in Ayurvedic medicine and has recently gained popularity as a dietary supplement for its various health benefits [104,105].

### 4.1. Metabolism and Weight Management

Like EGCG, forskolin aids in weight loss but through different mechanisms. It stimulates cAMP production within adipose cells, promoting fat breakdown and boosting metabolism. Increased cAMP levels activate protein kinase A (PKA), which phosphorylates hormone-sensitive lipase (HSL) and other enzymes involved in lipolysis, breaking down stored fat into free fatty acids and glycerol. This contributes to fat loss and increases metabolic rate [106,107]. Some clinical studies have shown that forskolin can reduce body fat, particularly in men [108]. In a study of overweight and obese men, forskolin reduced body fat percentage and fat mass while maintaining muscle mass. The study also suggested that forskolin might increase testosterone levels, benefiting muscle mass, energy levels, and hormonal balance, likely due to its role in cAMP elevation [109].

### 4.2. Cardiovascular Health

Forskolin has traditionally been used to manage cardiovascular health. It may lower blood pressure by relaxing blood vessels and improving blood flow, which can benefit those with hypertension [110]. Additionally, forskolin may enhance heart muscle contractility, useful for individuals with certain heart conditions, as cAMP plays a role in heart muscle contraction [111].

### 4.3. Respiratory Disorders

Regarding respiratory disorders, forskolin may help alleviate asthma by relaxing lung muscles, leading to bronchodilation, improving breathing, and reducing asthma attacks [112]. It may also reduce respiratory inflammation, benefiting people with asthma and other inflammatory conditions [113]. Furthermore, forskolin’s anti-inflammatory properties may reduce chronic inflammation, which is linked to various health issues [114]. Additionally, forskolin has antioxidant properties that help protect cells from oxidative damage [105].

### 4.4. Diabetes

In diabetes, forskolin may improve insulin sensitivity and regulate blood sugar levels. Some studies suggest it could aid in glucose metabolism, potentially benefiting people with type 2 diabetes [115,116]. Preliminary research also indicates that forskolin may have anti-cancer properties, inhibiting the growth of certain cancer cells, though further research is needed to confirm its role in cancer treatment [117,118].

### 4.5. Neuroprotection

Forskolin has demonstrated significant neuroprotective properties, primarily through the receptor-independent activation of adenylyl cyclase and subsequent elevation in cAMP levels. The increase in cAMP initiates downstream signaling pathways, including the activation of PKA and cAMP response element-binding protein (CREB), which is crucial for neuronal survival, differentiation, and plasticity [119,120]. Forskolin enhances the expression of neurotrophic factors such as brain-derived neurotrophic factor (BDNF) and its receptor TrkB, promoting RGC survival and neuronal resilience in neurodegenerative models [121,122]. Indeed, neurodegenerative diseases like Parkinson’s Disease (PD), multiple sclerosis (MS), and Alzheimer’s disease (AD) are major global health challenges due to their progressive nature and limited treatment options. Forskolin’s ability to modulate key neuroprotective mechanisms offers a promising approach to developing disease-modifying therapies.

PD is characterized by the loss of dopaminergic neurons in the substantia nigra, and reduced protein kinase A (PKA) signaling in the midbrain is implicated in PD pathogenesis. Forskolin has shown the capacity to restore PKA activity, reduce α-synuclein aggregation, and elevate levels of neurotrophic factors such as BDNF and nerve growth factor (NGF) [120,121]. The loss of dopaminergic neurons in PD leads to motor and non-motor impairments. Experimental models using the neurotoxin 6-OHDA mimic PD-like symptoms, including mitochondrial dysfunction, free radical formation, and CREB signaling disruption. In 6-OHDA-induced rat models, forskolin treatment (15–45 mg/kg orally) effectively reversed behavioral and neurochemical abnormalities. Behavioral tests, along with cellular and molecular analyses, confirmed forskolin’s role in restoring mitochondrial function and repairing neurodegeneration [123].

Intranasal delivery of forskolin combined with the nootropic agent Noopept (CNS/CT-001) in PINK1 knockout (KO) rat models of PD reversed motor deficits, hindlimb weakness, and neurodegeneration. These therapeutic effects were attributed to enhanced PKA signaling and the clearance of cortical α-synuclein aggregates, indicating forskolin’s potential as a disease-modifying agent [124].

Current treatments of PD, like Levodopa, alleviate symptoms but are not disease-modifying and have significant side effects. Forskolin, administered intraperitoneally, was evaluated again in the PINK1-KO rat model of PD. Results showed that forskolin reversed motor symptoms, restored hindlimb strength, prevented neuronal loss, enhanced brain energy production, and restored PKA activity, offering long-lasting effects (>5 weeks). Unlike Levodopa, which provides only temporary relief, forskolin demonstrated superior, disease-modifying potential for PD therapy [125]. Therefore, these findings underscore FSK’s potential as a preventive and therapeutic agent for PD via the AC/cAMP/CREB pathway activation.

Forskolin has also shown efficacy in models of multiple sclerosis, a condition involving oligodendrocyte loss and neuronal demyelination driven by mitochondrial dysfunction and inflammation. In experimental MS rat models induced with ethidium bromide, forskolin at doses of 40–60 mg/kg enhanced remyelination, improved motor and cognitive functions, and reduced levels of inflammatory cytokines. It also restored mitochondrial function and reduced oxidative stress, further validating its neuroprotective effects. In this case, the mechanisms underlying these effects were also primarily mediated through the activation of the AC/cAMP/CREB pathway, which restored mitochondrial function, enhanced neurotransmitter levels, and mitigated inflammation [126].

Forskolin has also demonstrated significant neuroprotective effects in Alzheimer’s disease (AD) models. AD is marked by the accumulation of amyloid-beta (Aβ) plaques, tau protein hyperphosphorylation, oxidative stress, and neuroinflammation, all contributing to neurodegeneration. Forskolin has shown promise in targeting these pathological mechanisms, providing a multifaceted approach to mitigating AD progression.

In transgenic APP/PS1 mice, oral administration of forskolin (100 mg/kg body weight) reduced Aβ plaque deposition in the cortex and hippocampus, critical regions for memory and cognition. This reduction was associated with improved behavioral outcomes, such as restored nest construction ability and sociability, suggesting a reversal of cognitive and social deficits. Furthermore, forskolin reduced neuroinflammatory markers, including transforming growth factor β, glial fibrillary acidic protein, and Iba-1, highlighting its role in regulating microglial and astrocytic activity [127]. The AC/cAMP/PKA pathway activated by forskolin also counteracts tau hyperphosphorylation and associated neurodegeneration. Studies in neuronal-like cells derived from neuroblastoma treated with forskolin demonstrated delayed ectopic cell cycle reactivation, a phenomenon linked to neuronal death in AD. Forskolin’s co-administration with PACAP further emphasized its regulatory role in maintaining neuronal integrity [128]. Additionally, forskolin has been implicated in enhancing lysosomal reacidification in astrocytes, which is crucial for Aβ degradation. By reversing lysosomal alkalization and increasing cathepsin D activity, forskolin facilitated the clearance of Aβ aggregates, thereby reducing amyloid burden and restoring cellular homeostasis [129]. These effects align with forskolin’s broader neuroprotective properties, including its ability to modulate oxidative stress and support mitochondrial function.

Research has also linked forskolin to improved synaptic function in AD models. Forskolin’s activation of the cAMP pathway prevented Aβ-mediated inhibition of long-term potentiation (LTP) in hippocampal neurons, a key mechanism underlying learning and memory. This effect, mediated through the activation of PKA, underscores forskolin’s potential in preserving synaptic plasticity in AD [130]. Furthermore, forskolin-induced cAMP elevation has been associated with increased secretion of non-amyloidogenic amyloid precursor protein (APPsα), which has neuroprotective effects and enhances memory. This dual role of forskolin in reducing amyloidogenic processes and promoting protective APP pathways positions it as a promising therapeutic agent for AD [131].

Collectively, these findings demonstrate forskolin’s capacity to address multiple pathological features of AD, including amyloid plaque accumulation, tau hyperphosphorylation, oxidative stress, neuroinflammation, and synaptic dysfunction. Its actions, primarily mediated through the AC/cAMP/PKA signaling pathway, highlight its therapeutic potential as a disease-modifying agent for AD. Continued exploration of its clinical applications is necessary to translate these preclinical findings into effective treatments.

Overall, Forskolin represents a novel therapeutic agent with significant potential in addressing the pathological mechanisms underlying neurodegenerative diseases. Its ability to confer long-lasting effects, particularly through non-invasive administration routes like intranasal delivery, underscores its suitability for further clinical development. Ongoing research should aim to optimize forskolin’s formulations and establish its efficacy and safety in human trials.

### 4.6. Anti-Inflammatory Antioxidant

Forskolin’s anti-inflammatory effects, combined with its ability to mitigate oxidative stress, contributes to its efficacy on neurodegenerative diseases.

Neuroinflammation is a hallmark of many neurodegenerative diseases, including autism, where activated microglia and astrocytes contribute to the inflammatory milieu. A recent seminal study found that forskolin effectively modulates these glial cells, reducing their activation [132]. This modulation leads to a decrease in the secretion of pro-inflammatory cytokines, which are typically elevated in neuro-inflammatory states. By dampening the inflammatory response, forskolin helps to create a more favorable environment for neuronal health and function. Additionally, the study highlights forskolin’s potent antioxidant effects. Oxidative stress is another crucial factor in the pathogenesis of neurodegenerative diseases. It results from an imbalance between the production of free radicals and the body’s ability to counteract their harmful effects. Forskolin’s ability to enhance the activity of antioxidant enzymes and reduce the levels of ROS underscores its protective role. This antioxidant action not only shields neurons from oxidative damage but also supports overall cellular homeostasis.

In an experimental model of MS induced by ethidium bromide (EB), a chemical that causes demyelination in rats, forskolin’s anti-inflammatory properties were pivotal to its effects [126]. By reducing the activation of microglia and astrocytes, which are involved in the inflammatory response, forskolin helped to create a more favorable environment for neuronal health and myelin repair. This anti-inflammatory effect, combined with forskolin’s ability to mitigate oxidative stress, contributed to its overall neuroprotective efficacy.

In a mouse model of cerebral amyloidosis, which is often used to study AD, forskolin-treated mice showed significant improvements in nest construction and sociability, indicating enhanced cognitive and social functions, which are often impaired in AD [127]. In this model, forskolin treatment led to a decrease in the expression of inflammatory markers such as transforming growth factor β, glial fibrillary acidic protein, and Iba-1 in the cortex. These markers are associated with the activation of microglia and astrocytes, which are involved in the inflammatory response in the brain. By reducing the activation of these glial cells, forskolin helps to mitigate neuroinflammation. The study also observed a reduction in the activation of microglia and astrocytes, suggesting that forskolin can regulate the inflammatory response mediated by these cells. This regulation is essential for maintaining a healthy brain environment and preventing further neuronal damage.

Overall, these studies provide compelling evidence that forskolin’s combined anti-inflammatory and antioxidant effects, along with its ability to improve mitochondrial function, confirming forskolin as a promising therapeutic agent for neurodegenerative diseases. By targeting multiple pathways and cellular processes, forskolin offers a multifaceted approach to neuroprotection, which could be beneficial in conditions such as autism, multiple sclerosis, and other neurodegenerative disorders.

## 5. Forskolin Efficacy on Retinal Diseases

Forskolin has shown promising therapeutic potential in the treatment of glaucoma and other retinal diseases, primarily through its effects on intraocular pressure (IOP) and neuroprotection.

### 5.1. IOP

In glaucoma, forskolin may be beneficial by reducing intraocular pressure (IOP), potentially preventing optic nerve damage and vision loss [133]. Elevated cAMP levels promote two primary effects: they stimulate the active resorption of the aqueous humor (AH) from the posterior chamber into the stroma [134] and cause relaxation of the ciliary muscle along with the trabecular meshwork [135]. This enhances AH outflow and effectively lowers intraocular pressure (IOP), a critical factor in glaucoma management [136]. Additionally, forskolin has neuroprotective effects by enhancing cAMP levels, which support neuronal survival and function, providing a safeguard against neurodegeneration in RGCs in glaucoma [137]. In fact, forskolin induces on the one hand the synthesis and the expression of BDNF by astrocytes [138] and endothelial cells [139] lining RGCs. On the other hand, it enhances the translocation of the cognate receptor TrkB to the cell membrane of RGCs [140]. Clinical evidence supports this neuroprotective role of forskolin, although in association with other molecules [133,141].

### 5.2. Neuroprotection

Although the topical administration of forskolin is well studied in preclinical models, oral delivery may offer broader therapeutic benefits [133,141]. Numerous experimental studies suggest forskolin as a neuroprotective agent due to its ability to reduce intraocular pressure (IOP) in both animal models and humans [134,142]. Its protective effects on RGCs in glaucoma models are also well documented [119], with indirect neuroprotection arising from its IOP-lowering capacity. In a double-blind, randomized clinical trial, patients with primary open-angle glaucoma treated with 1% forskolin eye drops (administered three times daily for 4 weeks) exhibited significant IOP reduction [143], possibly due to forskolin’s ability to reduce aqueous humor accumulation [144]. In another clinical study, a dietary supplement containing forskolin lowered IOP and improved pattern electroretinogram amplitude in glaucoma patients, suggesting enhanced RGC function or survival [145]. Forskolin’s neuroprotective effects are partially attributed to its role in boosting neurotrophin activity. Meyer-Franke et al. demonstrated that forskolin, when combined with brain-derived neurotrophic factor (BDNF), ciliary-derived neurotrophic factor (CTNF), and insulin-like growth factor-1 (IGF-1), increased RGC survival in vitro [119]. Similarly, in a feline model, forskolin augmented RGC survival when used alongside BDNF and CTNF following axonal injury [146]. Animal studies also support these findings. Dietary supplementation with forskolin, homotaurine, spearmint, and B vitamins protected RGCs from degeneration in rodent models of optic nerve injury [147] and glaucoma [148]. This combination decreased inflammation and apoptotic markers, preserving visual function without altering IOP in glaucomatous models [149].

Moreover, forskolin has also shown promising therapeutic potential in the treatment of glaucoma and other retinal diseases, primarily through its effects on oxidative stress, inflammation, blood perfusion and angiogenesis, and glucose metabolism.

### 5.3. Oxidative Stress and Inflammation

Forskolin’s antioxidant properties further protect ocular tissues from oxidative stress-induced damage, a common pathway in diseases like AMD and diabetic retinopathy [105]. Forskolin also helps reduce inflammation, which is a contributing factor in retinal diseases [150]. By lowering the levels of inflammatory factors such as ICAM-1 and TNF-α, and by decreasing the number of adherent leukocytes in retinal microvasculature, forskolin mitigates retinal inflammation, especially in diabetic conditions [114,151,152]. This reduction in inflammation protects retinal cells from further damage, particularly in RGCs, where forskolin’s neuroprotective effects play a crucial role [153].

### 5.4. Blood Perfusion and Angiogenesis

Forskolin promotes vasodilation by increasing cyclic AMP (cAMP) and cyclic GMP (cGMP) levels, improving blood flow and supporting the delivery of oxygen and nutrients to the retina [154]. This is particularly beneficial in conditions like AMD, where blood supply to the retina is often impaired. Additionally, forskolin inhibits angiogenesis, which is the abnormal formation of new blood vessels that contributes to vision loss in proliferative diabetic retinopathy and neovascular AMD [155]. In a preclinical experimental setting, it was investigated whether forskolin, a protein kinase A (PKA) agonist, affects Toll-like receptor 4 (TLR4) signaling and retinal endothelial cell (REC) permeability in high-glucose conditions [156]. The results showed that forskolin restored the levels of tight junction proteins (ZO-1 and occludin) and improved REC permeability. Both forskolin and TLR4 inhibition reduced the high-glucose-induced increase in REC permeability, though their actions were not synergistic. Forskolin influenced both MyD88-dependent and -independent TLR4 signaling pathways independently of Epac1. These findings suggest that targeting PKA or TLR4 could offer new therapeutic approaches for retinal vascular conditions. This may help reduce fluid accumulation and retinal edema. Diabetic retinopathy can involve the formation of new, abnormal blood vessels (neovascularization). If forskolin can influence pathways related to angiogenesis (the formation of new blood vessels), it could potentially play a role in preventing or reducing this process. Indeed, a study involving a cancer model showed that forskolin’s activation of the cAMP/PKA pathway could have an inhibitory effect on vascular endothelial growth factor (VEGF) production, a key driver of retinal angiogenesis in pathologies like diabetic retinopathy [157].

### 5.5. Glucose Metabolism

In diabetic retinopathy, forskolin plays a role in modulating glucose metabolism, which is crucial for preventing retinal damage. Studies have shown that forskolin can reduce retinal glucose concentrations by lowering the expression of glucose transporter 1, a protein that mediates glucose uptake into cells. This leads to a decrease in retinal glucose levels and reduces inflammation associated with diabetic conditions [158]. Forskolin’s ability to restore tight junction proteins and improve retinal endothelial cell permeability further supports retinal health by preventing fluid leakage and retinal edema, common issues in diabetic retinopathy.

Improving glucose metabolism is critical in the prevention and treatment of diabetic retinopathy, as prolonged hyperglycemia accelerates retinal damage through multiple pathways. Forskolin’s ability to regulate glucose levels and enhance retinal endothelial cell function offers a dual benefit of addressing both systemic metabolic dysregulation and localized retinal health. This metabolic regulation may provide a therapeutic advantage in managing the progression of retinal diseases.

**Figure 4:**
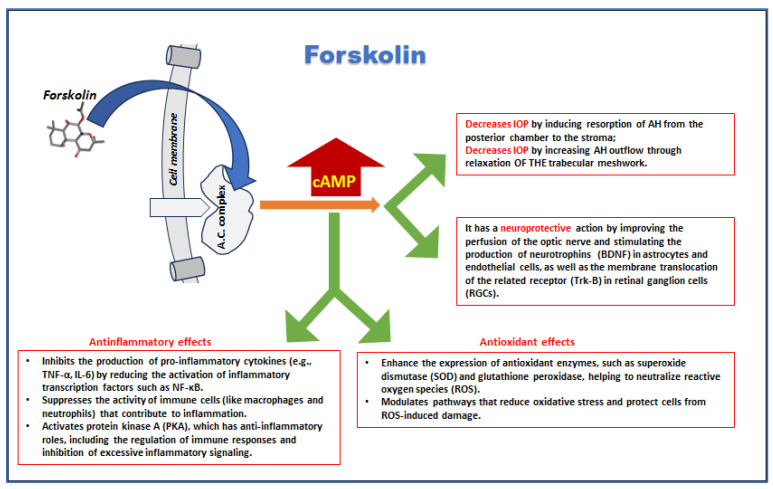
Multiple effects of forskolin (a receptor-independent activator of adenyl-cyclase) relevant to ophthalmic diseases.

Collectively, these biochemical properties position forskolin as a valuable molecule for developing treatments aimed at reducing IOP, protecting against oxidative and inflammatory damage, and preserving neuronal health in various eye diseases (Figure 4).

## 6. Predicted Cooperative Effects of EGCG and Forskolin

Although no experimental data have been published so far, the predicted cooperative effects of epigallocatechin gallate (EGCG) and forskolin in the treatment of glaucoma and other ophthalmic diseases stem from their complementary biochemical properties and mechanisms of action. EGCG, known for its potent antioxidant and anti-inflammatory properties, effectively reduces oxidative stress and inflammation, which are key contributors to the pathogenesis of several retinal diseases, including glaucoma, diabetic retinopathy, and AMD. In addition to reducing oxidative damage, EGCG’s neuroprotective capabilities are crucial in preserving RGCs, which helps prevent optic nerve damage and vision loss in glaucoma.

Forskolin, on the other hand, acts primarily by activating adenylate cyclase, an enzyme that increases cyclic AMP (cAMP) levels. This leads to enhanced aqueous humor outflow and a consequent reduction in intraocular pressure (IOP), a key therapeutic target in glaucoma management. By lowering IOP, forskolin directly addresses one of the main risk factors for optic nerve damage and disease progression in glaucoma. In clinical studies, forskolin has demonstrated its ability to act synergistically with other compounds to reduce intraocular pressure (IOP) and improve visual parameters in glaucoma patients [133,141]. This evidence supports the hypothesis that a combination of forskolin with EGCG could further enhance therapeutic outcomes, leveraging their complementary mechanisms of action to optimize retinal health and disease management.

Indeed, when combined, EGCG and forskolin may offer a multifaceted approach to ocular treatment. EGCG’s neuroprotective and anti-inflammatory actions help enhance the overall health of ocular tissues, protecting against retinal degeneration and inflammation, while forskolin’s IOP-lowering effect specifically targets glaucoma’s root cause. Furthermore, both compounds exhibit anti-inflammatory and antiangiogenic properties, which can be particularly beneficial in managing diabetic retinopathy and AMD, where abnormal blood vessel growth and inflammation exacerbate retinal damage.

The synergistic effects of EGCG and forskolin, therefore, hold the potential to not only slow disease progression but also improve overall ocular health by addressing multiple pathogenic mechanisms. Through their combined antioxidant, anti-inflammatory, neuroprotective, and IOP-lowering actions, these compounds can offer a comprehensive therapeutic strategy for retinal diseases.

Additionally, considering the well-established link between metabolic dysfunction and retinal diseases, the systemic effects of both EGCG and forskolin—especially their roles in glucose metabolism and weight management—add another layer of therapeutic potential. By improving metabolic health, these compounds could reduce the overall oxidative and inflammatory burden on the retina, potentially slowing the progression of conditions such as diabetic retinopathy and AMD. This predicted cooperative interaction between EGCG and forskolin suggests a promising avenue for future research and development of comprehensive treatment strategies for glaucoma and other ophthalmic diseases, leveraging the strengths of both compounds to achieve superior clinical outcomes.

## 7. Formulation Issues

Despite their efficacy, the oral bioavailability of both EGCG (epigallocatechin gallate) and forskolin faces significant challenges due to their poor solubility, limited permeability, and instability in the gastrointestinal tract [159]. EGCG, being a polyphenolic compound, suffers from poor aqueous solubility and is further hindered by its large molecular size and hydrophilicity, which restrict its absorption. It is also highly susceptible to degradation in the gut environment [160]. Encapsulation techniques, nanoparticles, and co-administration with substances like piperine or phospholipids have been explored to improve EGCG’s bioavailability [161,162]. Similarly, forskolin, a labdane diterpene, shares the problem of limited solubility and permeability, exacerbated by its hydrophobic nature and susceptibility to enzymatic breakdown [160]. To improve the bioavailability of these compounds, several advanced formulation strategies have been explored [163]. Nanoparticle-based delivery systems can enhance their solubility and stability, promoting better absorption. Self-emulsifying drug delivery systems (SEDDSs) can improve both solubility and permeability by increasing lipophilicity and facilitating intestinal transport. Complexation with suitable carriers is another strategy that can boost both solubility and stability, potentially improving the systemic exposure of these compounds [163]. Lipid nanoparticle encapsulation also enhances ocular retention, making it a promising topical delivery method for retinal diseases [164].

Taken together, these methods could significantly enhance the bioavailability of EGCG and forskolin, and co-administration may offer synergistic therapeutic effects in treating various conditions.

In summary, the bioavailability of EGCG and forskolin is hindered by their poor solubility, permeability, and stability. Nanoparticles, SEDDSs, and complexation are promising strategies to improve their oral bioavailability and might allow their co-administration, potentially offering synergistic benefits.

## 8. Conclusions

EGCG and forskolin hold substantial potential as therapeutic agents for retinal diseases, attributed to their combined antioxidant, anti-inflammatory, neuroprotective, and intraocular pressure (IOP)-lowering effects. Preclinical studies, particularly those conducted in vitro and in animal models, strongly support their efficacy in mitigating the progression of conditions such as glaucoma, age-related macular degeneration (AMD), and diabetic retinopathy (DR). However, translating these findings into clinical applications remains challenging due to several critical limitations.

One major limitation is the absence of robust clinical studies confirming the therapeutic effects of EGCG and forskolin in humans. While preclinical data provide a strong foundation, the lack of extensive clinical trials hinders the ability to validate these findings and adapt them for practical use. This represents a significant gap that must be addressed to move these compounds closer to real-world treatment strategies for retinal diseases.

Another critical challenge lies in the bioavailability of EGCG and forskolin. Both compounds exhibit poor solubility, limited permeability, and instability within the gastrointestinal tract, which can significantly reduce their effectiveness when administered orally. This bioavailability issue underscores the need for innovative formulation approaches to enhance their systemic exposure and therapeutic potential. Techniques such as encapsulation, nanoparticle-based delivery, and other advanced drug delivery systems must be further explored to address these pharmacokinetic limitations effectively.

This review also highlights the promising prospect of combining EGCG and forskolin for their potential synergistic effects. However, our understanding of how these compounds interact in vivo remains incomplete. Future research must focus on elucidating these interactions, optimizing dosages, and determining the mechanisms through which their combined effects can maximize therapeutic outcomes. Addressing these knowledge gaps will be essential for validating their role in retinal disease management.

Finally, while significant advancements have been made in preclinical research, the field urgently requires well-designed clinical trials to evaluate the safety and efficacy of EGCG and forskolin, both individually and in combination. These trials will be critical in determining whether the promising benefits observed in preclinical models can be replicated in human patients. Additionally, they would provide clarity on whether these compounds can serve as adjuncts or alternatives to conventional therapies.

In conclusion, EGCG and forskolin demonstrate strong preclinical potential, but substantial efforts are needed to overcome their bioavailability challenges, confirm their synergistic benefits, and validate their efficacy through comprehensive clinical studies. Bridging the gap between preclinical findings and clinical applications will be crucial in developing effective combination therapies, ultimately optimizing the benefits of these compounds for retinal health.

## 9. Future Directions and Requirements

To overcome the limitations outlined in this review, the following strategies can be suggested:

1. Initiate Phase I/II Clinical Trials: Launch small-scale clinical trials focusing on the safety, tolerability, and preliminary efficacy of EGCG and forskolin, both individually and in combination. These studies should prioritize enrolling participants with conditions like glaucoma, AMD, or DR to evaluate the real-world impact of these compounds in a controlled setting.
2. Develop Advanced Formulation Techniques: Address the bioavailability challenges of EGCG and forskolin by investing in advanced drug delivery systems. This could include encapsulation in liposomes, use of nanoparticle-based carriers, or the development of sustained-release formulations. These strategies could improve the compounds’ stability, solubility, and absorption, enhancing their therapeutic potential.
3. Explore In Vivo Synergistic Mechanisms: Design preclinical studies to deepen the understanding of how EGCG and forskolin interact in vivo. This would involve investigating their combined pharmacodynamics and pharmacokinetics, as well as their effects on specific retinal disease pathways. Results from these studies could guide dosing strategies for clinical trials.
4. Conduct Bioavailability Studies: Undertake pharmacokinetic studies to determine the optimal administration routes and dosages that maximize systemic exposure to both compounds. Exploring alternatives to oral delivery, such as ocular inserts or topical eye drops, could be particularly beneficial for targeting retinal diseases.
5. Engage in Multidisciplinary Collaborations: Foster collaborations among pharmacologists, ophthalmologists, and formulation scientists to accelerate the development of viable therapeutic options. Cross-disciplinary approaches can facilitate the integration of preclinical findings with clinical trial designs and advanced formulation technologies.
6. Secure Funding and Regulatory Support: Probably the most important and more difficult challenge because of the limited income expected from a food supplement will be to seek funding from public health organizations and private entities to support the transition from preclinical to clinical research. Early engagement with regulatory bodies could streamline the approval process for clinical trials.

By addressing these limitations, researchers can take critical steps toward translating the preclinical promise of EGCG and forskolin into effective, clinically validated therapies for retinal diseases.

## Data Availability

No new data were created or analyzed in this study.
